# *Abelmoschus esculentus* (L.): Bioactive Components’ Beneficial Properties—Focused on Antidiabetic Role—For Sustainable Health Applications

**DOI:** 10.3390/molecules24010038

**Published:** 2018-12-21

**Authors:** Alessandra Durazzo, Massimo Lucarini, Ettore Novellino, Eliana B. Souto, Patricia Daliu, Antonello Santini

**Affiliations:** 1 CREA-Research Centre for Food and Nutrition, Via Ardeatina 546, 00178 Rome, Italy; alessandra.durazzo@crea.gov.it (A.D.); massimo.lucarini@crea.gov.it (M.L.); 2 Department of Pharmacy, University of Napoli Federico II, Via D. Montesano 49, 80131 Napoli, Italy; ettore.novellino@unina.it (E.N.) patricia.daliu@unina.it (P.D.); 3 Department of Pharmaceutical Technology, Faculty of Pharmacy, University of Coimbra, 3000-354 Coimbra, Portugal; souto.eliana@gmail.com; 4 CEB-Centre of Biological Engineering, University of Minho, Campus de Gualtar, 4710-057 Braga, Portugal

**Keywords:** okra, plant components, interactions assessment, integrated food research, chemometrics, antidiabetic properties, applications

## Abstract

The main features of the okra, *Abelmoschus esculentus* (L.), are highlighted. The evaluation of interactions between biologically active compounds and other components of the food matrix can be considered as the first action in the investigation of potential benefits of this annual herb. Moreover, updated examples of current and innovative directions in an integrated and multidisciplinary approach are discussed, with particular attention to chemometrics. Among the main effects attributed to okra, its antidiabetic property is the focus. Finally, the use of okra in different fields will be discussed.

## 1. A Mini Overview of Okra *Abelmoschus esculentus* (L.) Features

One of current main challenge is promote and ensure healthy food in a sustainable manner, indeed the attention to less common food source is addressed. *Abelmoschus esculentus* L. (Moench) of the Malvaceae family (also known as *Hibiscus esculentus*) is an annual herb that is more commonly known in several other vernacular names as ladies finger, okra, bhindi, or gumbo [1,2,3]. This important vegetable crop, native to Africa, is grown in tropical, subtropical, and warm temperate climates in different countries from Africa to Asia, Southern Europe, and America [4,5,6].

The okra fruit/pod is a greenish capsule with length of 10–30 cm long and a diameter of 1–4 cm, it is slightly curved, tapers to a blunt point, a six-chambered pod of fibrous texture, and contains numerous seeds [7].

Okra is known for its good palatability among different regions and its culinary uses are wide. Its immature, fresh, green seed pods are eaten as vegetable, while the extract obtained from the fruit is used in different recipes thicken stews, soup, and sauces to increase their consistency [8,9]. It offers, in fact, a mucilaginous consistency after cooking. The immature pods are also used in making pickle. Often water-soluble polysaccharides from okra are also used in ice-cream, potato chips, and baked goods, providing a healthy option and more stable shelf-life [10,11,12,13,14].

## 2. Biologically Active Components in Okra: Study Approach and Current and Innovative Directions

### 2.1. Main Plant Compounds and Their Interactions Assessment

Generally, okra is a high-value crop because it represents a source of nutrients that are important to human health, e.g., vitamins, potassium, calcium, carbohydrates, dietary fiber, and unsaturated fatty acids such as linolenic and oleic acids, and also of bioactive chemicals [15,16]. Okra is a multipurpose crop due to the varied use of its leaves, buds, flowers, pods, stems, and seeds [17]. Okra has long been used as a vegetable and a source of dietary medicine [18,19,20,21]. Indeed, beside its nutritional role, it is suitable for certain medical and industrial applications [19].

The profile of the bioactive components in different parts of okra is well documented: for okra pod polyphenolic compounds, carotene, folic acid, thiamine, riboflavin, niacin, vitamin C, oxalic acid, and amino acids [21,22,23,24]; for okra seed polyphenolic compounds, mainly oligomeric catechins and flavonol derivatives, protein (i.e., high lysine levels), and oil fraction (in particular, its derived oil is rich in palmitic, oleic, and linoleic acids) [25,26,27,28,29,30,31,32]; for root carbohydrates and flavonol glycosides [33], and manly minerals, tannins, and flavonol glycosides for leaves [34,35].

Liao et al. [36] described the presence in different percentages of the total phenolics and total flavonoids and antioxidant properties in different part of plant i.e., flower, fruit, leaf, and seed.

Many of components (flavonoids, polysaccharides, and vitamins) of okra have been proved to possess significant biological activities [37,38,39,40,41,42]. The assessment of interactions of bioactive components throughout the measure of antioxidant properties [42] represents a first step for the comprehension of their biological activities and beneficial properties.

Currently, the main approaches of investigations [43] of bioactivities interactions in food matrices can be identified as follows: (1) development of the model system of interactions [44,45,46,47]; (2) distinction of extractable and non extractable compounds [48,49,50]; and (3) study of biologically active compounds-rich extracts [51,52].

Several studies reported antioxidant properties of okra [26,27,29,30,36,53,54,55,56]. For instance, Geng et al. [54] studied the extraction and antioxidant activity of phenolic compounds from okra flowers: the extract exhibits a strong DPPH radical scavenging activity and reducing power, which makes it a potential functional ingredient in the food and pharmaceutical industries. The work of Hu et al. [30] determined the antioxidant activity of extract and its major constituents identified, i.e., quercetin 3-*O*-glucosyl (1→6) glucoside (QDG) and quercetin 3-*O*-glucoside (QG), from okra seed on oxidative stress induced by carbon tetrachloride (CCl_4_) in a rat hepatocyte cell line: both total extracts and total constituents exhibited excellent reducing power and free radical scavenging capabilities including DPPH, superoxide anions, and hydroxyl radicals. Moreover the same authors [30] showed that total phenolic extract (TPE), QG, and QDG pretreatments significantly alleviated the cytotoxicity of CCl_4_ on rat hepatocytes, with attenuated lipid peroxidation, increased SOD and CAT activities, and decreased GPT and GOT activities. The recent work of Graham et al. [55], by studying the total phenol content and antioxidant activity of okra seeds from different genotypes, marked how the utilization of okra seeds as tea or in other diets could provide antioxidant benefits. Concerning oil from okra seed, Dong et al. [29] showed the DPPH radical scavenging activity of the oil extracted by screw press expression methodology is higher than those by supercritical carbon dioxide extraction and solvent extraction procedure.

### 2.2. Integrated Research, Emerging Technologies, and Chemometrics

Recently, studies on the evaluation of bioactive components are addressed to combine emerging analytical technologies and rapid and green procedures, with the statistical methods like the chemometrics science in a multidisciplinary approach.

Among these, the use of the spectroscopic technique coupled with statistical methods are being applied for the study compositions in nutrients and bioactive compounds. An example of isolation and standardization fractions with specified bioactivities assessed by Fourier transform infrared spectroscopy (FTIR) is given by Doreddula et al. [57]; the authors highlighted differences in the FTIR spectrum of aqueous and methanolic seed extracts of *Abelmoschus esculentus*: FTIR spectra revealed the presence of various functional groups such as alkyl, ketone, aldehyde, carboxylic acids, esters, and amide in the aqueous and methanolic extracts of *Abelmoschus esculentus*, respectively.

The recent work of Xia et al. [58] developed a rapid method based on Fourier transform near infrared (FT-NIR) spectroscopy for analysis of antioxidant compounds and activity of okra seeds. Another example was given by Zhang et al. [59] that used near-infrared (874–1734 nm) hyperspectral imaging technology combined with chemometrics to identify parental and hybrid okra seeds.

The current research of Wang et al. [60] determined the optimal conditions for ultrasound-assisted extraction of a water-soluble polysaccharide, raw okra polysaccharide (ROP), from the fruit of okra using response surface methodology: with the optimal condition and purification using a DEAE-Sepharose Fast Flow column and Sepharose CL-6B column three elution peaks—ROP −1, −2, and −3—were isolated. The authors identified and characterized the primary structural features (glucose, mannose, galactose, arabinose, xylose, fructose, and rhamnose) and molecular weight (1.92 × 105 Da) of ROP-2 the peak with the highest yield, by gas chromatography, Fourier transform infrared spectroscopy, and high-performance liquid chromatography [60]. Moreover, as reported by the authors [60], superoxide radical scavenging assay and DPPH radical scavenging assay further revealed the significant in vitro antioxidant activity of ROP-2.

It is worth mentioning the work of Pande et al. [61] that reported an innovative utilization of aqueous extract of *Abelmoschus esculentus* (okra) in an environmental friendly method to synthesize silver nanoparticles. The biosynthesis involved reducing the number of silver ions in the solution of silver nitrate with an aqueous vegetable extract of *Abelmoschus esculentus*, was monitored by FTIR. Several authors characterized the okra mucilage for studying its use in the biofilm [62,63]. 

## 3. An Updated Overview of Potential Beneficial Effects Associated to Okra: Focus on Antidiabetic Properties

Generally, plants with medicinal values are known to play significant role globally particularly in the management and treatment of various chronic diseases. Potential beneficial effects are associated to okra and their components cardioprotective, renal protective, neuroprotective, anticancer, analgesic, antiulcer, antibacterial, and antifatigue [64,65,66,67,68,69,70,71]. It is worth mentioning the current review of Islam [72], which summarizes the phytochemical reports and biological activities of *Abelmoschus esculentus* L. from the database sources. The same author [73], together with others, reviewed the role of active constituents such as pectin, epigallocatechin, and quercetin of *Abelmoschus esculentus* (okra) on tumor biology, with attention to its role in tumorigenesis and future research prospect in treating cancer.

Focus here is the role of okra in the management and treatment of diabetes. Diabetes is a chronic health disease attributed to risk factors like obesity, physical inactivity, ageing, and genetic predisposition; in particular diabetes mellitus is a progressive metabolic disease and it has affected considerable percentage of population throughout the world, associating with considerable morbidity and mortality [74].

### Evidences of Current Research

Dubey and Mishra [75], in a recent review, studied the role of okra in treatment of diabetes, with particular regards to the role of okra in lowering the glucose level; the authors underlined how the ability in managing increased blood glucose concentration of okra seed was known in traditional medicine and, along with years of modern research, has correlated this traditional claim with scientific evidences. Among the studies reported by Dubey and Mishra [75], Sabitha et al. [76] showed how the administration of peel and seed powder of *Abelmoschus esculentus* at 100 and 200 mg/kg dose in treptozotocin (STZ)-induced diabetic rats showed a significant reduction in blood glucose level and an increase in body weight than diabetic control rats; the same authors [77] in another work showed appreciable in vitro α-glucosidase and α-amylase enzyme inhibitory effects in aqueous extracts from both the peel and seed of *Abelmoschus esculentus* (L.) Moench. Another recent review on role of okra in prevention and management of diabetes and diabetic induced hyperglycemia is carried out by Khosrozadeh et al. [78]; the authors [78] marked how different studies showed that *Abelmoscus esculentus* (AE)/okra extract has a hypoglycemic effect that helps decrease blood glucose level. In addition, it leads to inhibition of cholesterol absorption and subsequently decreases the level of lipid and fat in the blood.

Some authors, in order to understand which chemical plays an important role in the antidiabetic activity of okra, are focused on study of okra polysaccharide and related characteristics. The structure of pectin in okra have investigated in several reports [79,80,81]. The recent work of Xu et al. [81] reported molecular characteristics and rheological properties of water-extractable polysaccharides derived from okra (*Abelmoschus esculentus* L.): the proportions of galacturonic acid and monosaccharides in the refined polysaccharides were 62.47% and 13.47%, respectively. Some reports have mentioned that okra polysaccharides are associated to several bioactivities linked to diabetes management, i.e., hypoglycemic activity, and are indicated as novel immunomodulators [37,82,83], in addition to others [84,85,86,87,88]. Fan et al. [37] reported that okra polysaccharide can lower body weight and glucose levels, improve glucose tolerance, and decrease total serum cholesterol levels in high-fat diet-fed C57BL/6 mice: okra polysaccharide regulated the gene expression of liver X receptors (LXRs) and peroxisome proliferator-activated receptors (PPARs) and their target genes in the liver and in the adipose tissue of the mice; the same authors [37] suggested that okra polysaccharide may have therapeutic effects on metabolic diseases via the inhibition of LXR and PPAR signaling. Chen et al. [83] reported that okra polysaccharide can increase the spleen index, splenocyte proliferation, and cytokine secretion in vivo. Moreover, Peng et al. [89] described the effects of okra water extracts as an adjuvant for diabetic nephropathy.

In particular, to understand which chemical plays an important role in the antidiabetic activity of okra, Liu et al. [82] purified and identified the dominant polysaccharide of okra as rhamnogalacturonan. The same authors [82] have documented the hypoglycemic effect of rhamnogalacturonan in vivo: by comparing with model group, the high-dose rhamnogalacturonan group showed decreased blood glucose level and glucose tolerance. The authors also underlined that further works are needed for the comprehension of hypoglycemic mechanism of rhamnogalacturonan [82].

Others were addressed towards the involvement some of quercetin derivatives [90,91]—well-known antioxidants [92]. Thanakosai & Phuwapraisirisan [90] describe two flavonolglucosides—isoquercetin and quercetin-3-*O*-β-glucopyranosyl-(1′′→6′′)-glucoside—in okra seeds as α-glucosidase inhibitors.

It is worth mentioning the recent review of Prahume et al. [93] that describes the state-of-art potential of *Abelmoschus esculentus* as a dietary agent in the treatment of diabetes induced hyperglycemia. In particular, Prahume et al. [93] marked how animal studies which involve *Abelmoschus esculentus* were statistically able to prove the blood glucose lowering effect in STZ induced diabetic rats [37,76,94,95,96,97].

As reported in Table 1, some examples of current researches in animal models are moving in this direction [91,98,99,100,101,102].

For instance, Abi et al. [98] concluded that whole extracts of okra are beneficial for a reduction in blood glucose when compared to a routine hypoglycemic agent or other okra extracts. Anjani et al. [91] showed that intervention of okra extract based on quercetin compound showed antihyperglycemic potential and improved malondialdehyde level in the streptozotocin-induced diabetic rat.

An interesting aspect was investigated by Muhammad et al. [99] who studied the antidiabetic effects of different parts i.e., whole okra, okra peel, and okra seed of one of the varieties of okra ex-maradi in alloxan-induced diabetic rats: all parts of the okra fruits showed significant (*p* < 0.05) reduction in blood glucose level, glycated hemoglobin, and improvement in lipid profile compared with the diabetic non treated control and comparable with metformin positive control [99]. A further work of the same authors proposed a possible mechanism of action of ex-maradi okra fruit variety (*Abelmoschus esculentus*) on alloxan-induced diabetic rats, by indicating that various parts of okra fruit have ability to stimulate glycogen synthesis in the liver and delay intestinal absorption of glucose with very significant glucose dialysis retardation index and high glucose adsorption capacity [100]. Moreover, the authors reported how histological examination of the pancreatic tissue after administration of okra fruit revealed evidence of pancreatic islets cells regeneration [100].

The effect of boiling and roasting on the antidiabetic activity of *Abelmoschus esculentus* (okra) fruits and seeds in type 2 diabetic rats was currently investigated by Nguekouo et al. [101]: boiling and roasting do not significantly influence the antidiabetic potential. This research underlines how appropriate processing methods or culinary form should be established in order to increase the health-promoting properties of this plant [101].

The current work of Majd et al. [102] evaluated the effects of okra powder on pancreatic islets and its action on the expression of PPAR-γ and PPAR-α genes in pancreas of high-fat diet (HFD) and streptozotocin-induced diabetic rats: downregulation of PPARs genes in the pancreas of diabetic rats after treatment with okra, demonstrates that okra may improve glucose homeostasis and β-cells impairment in diabetes through a PPAR-dependent mechanism.

It is worth mentioning a study by Moise et al. [103] based on cross-sectional design among African type 2 diabetics; the results showed how regular intake of Mediterranean diet, *Brassica Rapa*, beans, *Abelmoschus*, and *Musa acuminate* may significantly reduce the risk of blindness or its major causes [103].

Moreover, the current work of Muhammad et al. [104] has developed and tested a potential okra-based antidiabetic nutraceutical formulation from *Abelmoschus esculentus* (L.) Moench (ex-maradi variety): this formulation consists of a 10:90% (seeds:peel) formulation and has shown a reduction effect the rate of glucose adsorption and diffusion and thus can retard increase in postprandial blood glucose level than the other combinations tested in the study (proportions of the powdered seeds and peels samples 80:20%, 70:30%, 60:40%, 50:50%, and vice versa).

## 4. Use and Applications of Okra

Besides nutraceutical applications [105,106] in the prevention and/or therapy of some pathological health conditions, the entire plant is used to have several food and non food applications. Regarding the use of okra component as food ingredients, Qasem et al. [107] have evaluated the effect of okra gum on pasting and rheological properties of cake batter: the addition of okra negatively affected the viscous and thermal stabilities of cake batters, but increased the soluble fiber of the final product. Another example in this context is given by Romanchik-Cerpovicz et al. [108], who analyzed gluten-free rice flour tortillas prepared with okra gum: okra gum improved the texture in gluten-free, rice flour tortillas.

Moreover Farooq et al. [109] investigated the extraction and characterization of okra mucilage as pharmaceutical excipient to formulate solid oral dosage form: acceptable pH and organoleptic properties showed that it can be easily used to formulate various dosage form.

The bioenergetic uses of okra are discussed by Moosavi et al. [110]. De Alvarenga Pinto Cotrim et al. [62] investigated the development of edible films obtained from the mucilage of rejected okra fruits. Another interesting application of the use of okra mucilage for biofilms was shown in the current work of Araújo et al. [63]: okra mucilage/corn starch films for food applications were developed by casting and then characterized to establish its main requirements for packing material. Lee et al. [111] proposed an extraction from a plant-based bioflocculant—Hibiscus/*Abelmoschus esculentus* (okra)—using an environmentally friendly and economically feasible process; the authors marked how okra bioflocculant could offer a feasible and sustainable alternative to synthetic flocculants for water treatment and sludge dewatering applications due to its high efficiency in flocculating and dewatering, and can be extracted using only water as a solvent, minimizing the environmental footprint of the extraction process [111].

Finally, it is worth mentioning the work of Gogoi et al. [112] on utilization of agro-waste okra and its potentiality: fiber was extracted from the waste stem of the okra plant and was utilized for making different utility products to fulfill day today needs, i.e., ropes, paper, cleaning brushes, painting brushes, etc.

## 5. Conclusions

Generally evidence of the antidiabetic properties of okra and its oligosaccharides is achieved throughout several researches, even if full elucidation of the mechanism is needed in further studies. Few studies on other related components such as quercetin are present in the literature. Further studies should be addressed in the direction of developing functional foods, nutraceuticals, or drugs from okra components.

Moreover, the overarching goal is to carry out clinical trials addressing the formulation of new nutraceuticals as alternatives for diabetes management.

## Figures and Tables

**Table 1:** Some examples of current researches in potential antidiabetic in animal model.

Animal Models	Treatment	Studied Components of Okra Extract or Material Supplemented	Main Targeted Results	Ref.
Alloxan-induced diabetic Wistar rats	Animals were administered *Abelmoschus esculentus* peel (AEP), *Abelmoschus esculentus* seed (AES), and *Abelmoschus esculentus* seed and peel (AESP), all at 100 mg/kg and distilled water for the control. The last group had metformin at 100 mg/kg. Blood glucose was measured using the on days 5, 10 and 15.	No data accessible	The *Abelmoschus esculentus* peel, *Abelmoschus esculentus* seed, and *Abelmoschus esculentus* seed and peel (AESP) groups showed a significant decrease in blood glucose (*p* < 0.05) compared to the metformin group. AESP most significantly (*p* < 0.05) reduced blood glucose (96.84 ± 9.09) compared to metformin group (182.70 ± 34.81) on day 15.	[98]
Alloxan-induced diabetic rats	The rats were randomly divided into four large groups: (Whole Okra (WO), Okra Peel (OP), and Okra Seed (OS) and Control group (C)). Each one in subgroups based on dose of 100, 200, and 300 mg/kg/day. The control groups were Metformin (MC) (500 mg/kg), Diabetic (DC), and Normal (NC) Control groups. After a day of treatment blood samples were collected.	No data	All parts of the okra fruits (WO, OP, and OS) showed significant (*p* < 0.05) reduction in blood glucose level, glycated hemoglobin and improvement on lipid profile compared with the diabetic nontreated control and comparable with metformin positive control.	[99]
Alloxan-induced diabetic rats	24 Diabetic rat and eight normal rats were grouped as follows: -Normal rats Fasted (NF); -Diabetic rats Fasted (DF); -Normal rats, fasted and re-fed, untreated (NFRU); -Diabetic rats, fasted and re-fed, untreated (DFRU); -Diabetic rats, fasted, re-fed, and treated with 300 mg/kg Whole Okra fruit (WO); -Diabetic rats, fasted, re-fed, and treated with 300 mg/kg Okra peels (OP); -Diabetic rats, fasted, re-fed and treated with 300 mg/kg Okra seed (OS); -Diabetic rats, fasted, re-fed, and treated with 500 mg/kg metformin.	No data	Various parts of okra fruit have the ability to stimulate glycogen synthesis in the liver and delay intestinal absorption of glucose with very significant glucose dialysis retardation index (GDRI) and high glucose adsorption capacity (GAC). Histological examination of the pancreatic tissue after administration of okra fruit revealed evidence of pancreatic islets cells regeneration.	[100]
Streptozotocin and high-fat diet-induced type 2 diabetes Wistar rats	Animals were randomly assigned to six groups of 10 rats each, and treated for 28 days with either metformin or suspensions of one of the following, untreated fruits (UTF), boiled fruits, untreated seeds, and roasted seeds. Controls were made up of untreated non diabetic (T−) and diabetic (T+) animals. Fasting blood glucose was measured on a weekly basis.	Fiber Total phenolic content Free radical scavenging activity (DPPH)	Daily administration of processed and UTF and seed suspensions significantly decreased (*p* < 0.05) the blood glucose level of rats. Boiling and roasting do not significantly influence the antidiabetic potential of *A. esculentus* fruits and seeds.	[101]
Streptozotocin-induced diabetic rats	Animals were divided into six groups: normal control (N); diabetic control (DM); diabetic treated with green okra extract with the dosage of 5 mg/kg BW quercetin (GOE I) and 10 mg/kg BW quercetin (GOE II); diabetes treated with purple okra extract with the dosage of 5 mg/kg BW quercetin (POE I) and 10 mg/kg BW quercetin (POE II). The GOE and POE dissolved with Twin 1% were administered orally to the treatment group animals for 14 days.	Total Phenolic Quercetin	Administration of GOE I, GOE II, POE I, and POE II in diabetic rats showed significant (*p* < 0.05) reduction in blood glucose level (115.25 mg/dL; 86 mg/dL; 180.75 mg/dL; 91 mg/dL) and improve level of malondialdehyde.	[91]
High Fat Diet (HFD)/Streptozotocin(STZ)-induced diabetic rats	Animals were randomly divided into five equal groups as follows, group I: rats were fed with standard diet, group II: HFD-STZ-induced diabetic rats, group III: HFD-STZ-induced diabetic rats received *A. esculentus* (200 mg/kg). The *A. esculentus* powder was mixed with normal diet and administrated orally. Group IV: HFD-STZ-induced diabetic rats received metformin (200 mg/kg); group V: rats received normal diet and *A. esculentus* (200 mg/kg). Groups II, III, and IV were fed with HFD for four weeks, whereas groups I and V consumed normal diet during the same period.	Total phenolic content (extract); Total flavonoid content (extract); carbohydrate, protein, ash (dried plant).	Okra supplementation significantly decreased the elevated levels of FBS, total cholesterol, and TG and attenuated the homeostasis model assessment of basal insulin resistance (HOMA-IR) index in diabetic rats. The expression levels of PPAR-γ and PPAR-α genes that were elevated in diabetic rats, attenuated in okra-treated rats (*p* < 0.05).	[102]
